# Report on the American Association of Medical Physics Undergraduate Fellowship Programs

**DOI:** 10.1120/jacmp.v14i1.4159

**Published:** 2013-01-07

**Authors:** Jennifer B. Smilowitz, Stephen Avery, Paul Gueye, George A. Sandison

**Affiliations:** ^1^ Departments of Medical Physics and Human Oncology The University of Wisconsin Madison WI; ^2^ Department of Radiation Oncology Perelman Center for Advanced Medicine, University of Pennsylvania Philadelphia PA; ^3^ Physics Department Hampton University Hampton VA; ^4^ Department of Radiation Oncology The University of Washington Seattle WA USA

**Keywords:** medical physics education, summer undergraduate fellow, recruitment

## Abstract

The American Association of Physicists in Medicine (AAPM) sponsors two summer undergraduate research programs to attract top performing undergraduate students into graduate studies in medical physics: the Summer Undergraduate Fellowship Program (SUFP) and the Minority Undergraduate Summer Experience (MUSE). Undergraduate research experience (URE) is an effective tool to encourage students to pursue graduate degrees. The SUFP and MUSE are the only medical physics URE programs. From 2001 to 2012, 148 fellowships have been awarded and a total of $608,000 has been dispersed to fellows. This paper reports on the history, participation, and status of the programs. A review of surveys of past fellows is presented. Overall, the fellows and mentors are very satisfied with the program. The efficacy of the programs is assessed by four metrics: entry into a medical physics graduate program, board certification, publications, and AAPM involvement. Sixty‐five percent of past fellow respondents decided to pursue a graduate degree in medical physics as a result of their participation in the program. Seventy percent of respondents are currently involved in some educational or professional aspect of medical physics. Suggestions for future enhancements to better track and maintain contact with past fellows, expand funding sources, and potentially combine the programs are presented.

PACS number: 01.10.Hx

## I. INTRODUCTION

The American Association of Physicists in Medicine (AAPM) recognizes the importance of attracting a diverse group of academically talented and ambitious undergraduate physics students into the graduate studies and careers within medical physics. To this end, the AAPM supports two undergraduate fellowship programs: the Summer Undergraduate Fellowship Program (SUFP) and the Minority Undergraduate Summer Experience (MUSE). Both programs are managed by separate but overlapping subcommittees operating under the Education and Training of Medical Physicists Committee of the Education Council of the AAPM. Throughout the history of the programs there have been 28 unique AAPM members volunteering to serve on either, or both, subcommittees. SUFP fellows are matched with professional medical physics mentors in clinical, research, and combined settings. The MUSE program, while similar to SUFP, specifically targets applicants from underrepresented groups.

Although detailed statistics on medical physics graduate education (application, matriculation, and graduation) are difficult to obtain, there are indications of a significant increase in enrollment in graduate medical physics education programs. From 2001 (the year of the first SUFP fellowships) to 2012, the number of Commission on Accreditation of Medical Physics Education Programs (CAMPEP) accredited programs increased from eight to 38. According to a study of Medical Physics Graduate Programs conducted by CAMPEP and the Society of Directors of Academic Medical Physics Programs (SDAMPP), the number of applicants (and matriculated students) increased from 1351 (and 196) to 1668 (and 276) from 2008 to 2009. This same study indicated there were 237 graduates in 2010 from the 30 programs responding to the survey. Although limited to clinical professions, another measure of the growth of the field is the number of physicists seeking American Board of Radiology (ABR) certification. In 2006, 371 physicists initiated the process by taking the general section of the exam and, by 2010, that number had increased to 506.

A 2010 Workforce Study of Medical Physicists in the United States suggested that the number of board‐eligible new graduates required to meet the demand for medical physics services was projected to increase and a shortage could occur as early as 2017.^(^ ^1^ ^)^ A supply and demand model of the minimum number of new medical physicists required to meet the workforce demand in 2020 is estimated to be 125 per year.^(^ ^2^ ^)^ While there is some degree of uncertainty in the number of new medical physics graduates needed to meet the demand, it is clear that the field is growing. Incidentally, the field of medical physics can only benefit from attracting the most talented undergraduate students into graduate medical physics education.

It is crucial to identify, expose, and retain talented students early in their education to promote high‐quality medical physics graduate students. One exposure path is through summer programs targeted to physics undergraduates. Anecdotally, the professional and personal benefits of undergraduate research experience (URE) are widely acknowledged and evidenced by the large number of URE participants across many disciplines. However, studies evaluating the programs and tracking the outcomes are uncommon. Studies of undergraduate fellows and their faculty mentors report that the experience is overwhelmingly positive and enhances graduate school preparation and research skills, helps clarify postgraduate academic plans, and improves communication skills.^(^ ^3^ ^,^ ^4^ ^)^ The Survey of Undergraduate Research Experiences (SURE) is a quantitative study assessing the outcomes of URE as an educational tool and a means to encourage careers in science and technology. The SURE confirms the benefits of UREs, and reports the majority of participants (83% of 1135 students) began, or planned, postgraduate science education.^(^ ^5^ ^,^ ^6^ ^)^

A separate study funded by the National Science Foundation (NSF) concludes that participation in hands‐on research opportunities increased undergraduates' interest in the STEM fields (science, technology, engineering and mathematics). The study found that the early and enthusiastic involvement of undergraduates in research has the most effect in encouraging these students to pursue advanced degrees.^(^ ^7^ ^)^ The NSF indirectly funds undergraduates through its “Research Experience for Undergraduates” programs. However, a search of the current NSF awards with an undergraduate research component did not reveal any medical physics topics. The two AAPM sponsored programs (SUFP and MUSE) are the only medical physics‐specific programs, to the best of the authors' knowledge.

The SUFP was established in 2000 to provide URE opportunities specifically in medical physics. The first fellowships were awarded the summer of 2001. In 2006 the Minority Recruitment Subcommittee created MUSE as part of its efforts to target and recruit underrepresented students into medical physics. In general, MUSE provides fellows an experience that strongly emphasizes mentored clinical skills development, whereas the SUFP program incorporates a greater focus on summer research experience. Each program runs annually for ten weeks over the summer, and the students are paid a stipend to cover their living expenses. To attract undergraduate students from many subdisciplines of physics, solicitation for SUFP and MUSE applications are published in *Physics Today*. To generate further interest in the programs, emails are sent to undergraduate societies (Society of Physics Students, Health Physics Society, American Nuclear Society, and Biomedical Engineering Society). To increase awareness amongst AAPM members, SUFP — and later MUSE — have been highlighted in AAPM newsletters. Mentor participation for both SUFP and MUSE is voluntary and unpaid. The mentors must be full AAPM members. Research and clinical projects from any medical physics area are acceptable.

The objective of this article is to report on the history, participation, status, and impact of the programs after a decade of operation for SUFP and six years for MUSE. A summary of survey responses from previous fellows is presented. The scope is limited to recruitment of outstanding students into medical physics graduate programs through both the SUFP and MUSE programs, independent of minority status. The efficacy of the programs is assessed by four metrics: entry into a medical physics graduate program, board certification, publications, and AAPM involvement.

## II. MATERIALS AND METHODS

Data were compiled from fellow and mentor applications from 2001 to 2012 to generate statistics on the program participation. The data sources were paper files retained at the AAPM headquarters and, for later years, electronic files available online. The data from 2012 only were considered for calculation of the number of applicants and fellows. The data for all other years were used in the analysis of grade point averages, gender, host institution location, and type of internships supported.

In addition to applications, results from surveys distributed to both fellows and mentors at the end of each summer session up to and including 2011 were tallied. (Questions and results are summarized in Table 1.) The surveys ask fellows and mentors to rate the research experience and the administrative aspects of the program. The survey asks about the housing and travel experience and the length of the program. The questions are rated 1–4, with 1 signifying complete agreement with the statement and 4 signifying complete disagreement. Fellows are asked if a paper, report or presentation resulted from the fellowship. Fellows are encouraged to also include their contact information. Survey completion for fellows and mentors was neither anonymous nor mandatory. Therefore, only qualitative general conclusions are taken from these responses.

**Table 1:** Summary of End of Summer surveys.^a^

*Question*, 1=completely agree, 4=completely disagree *(Wording for mentor questions are listed in parenthesis)*	*Ave. Fellow Response*	*Ave. Mentor Response*
The process of contacting and dealing with the AAPM to indicate your desire to participate (that a summer opportunity) in a summer research experience was easily accomplished.	1.1	1.2
The process of initial communication with your selected institution (candidate) was easily accomplished.	1.1	1.3
Integrating yourself (the candidate) into the research/clinical environment was easily accomplished.	1.3	1.1
Assistance for securing housing was satisfactory.	1.7	1.6
The length of time was appropriate.	3.2	1.2
You (the mentor/institution) were (was) satisfied with your experience.	1.1	1.2
*Fellow only questions:*		
The institutional mentoring provided was satisfactory.	1.1	–
The research and/or clinical experience was interesting and rewarding.	1.2	–
As a result of this experience you would pursue a career in medical physics.	1.5	–
*Mentor only questions:*		
Please rate the skill level of the candidate (1 highest).	–	1.2
The research/clinical output of the candidate was sufficient.	–	1.1
The institution would participate in this experience again.	–	1.1

a 42 fellows responses and 32 mentors responses.

As part of this current report, the authors created an additional survey to be distributed to past fellows. The primary objective of the survey was to evaluate the effect of MUSE and SUFP on past recipients' entry into medical physics graduate programs. The objective of the survey was limited to quantifying the influence their participation in SUFP or MUSE had on the fellows' education pursuits, board examination status, AAPM involvement, and publications. It did not ask about the endpoint career outcomes. Understanding that many decisions are made between undergraduate summer experience and ultimate career choices and that many fellows are still in graduate school, the focus of the survey was on immediate education outcomes and activities. The AAPM Education Council reviewed the survey prior to distribution. The survey was generated using “Question Pro” and was administered by Ms. Jacqueline Ogburn, AAPM Education Manager. Approximately 70% of past applicants had current email addresses on file with the AAPM headquarters. An Internet search for the emails of the remaining students was performed. Of the 140 past fellows from 2001–2011, there were only 23 email addresses that could not be located. The survey was sent out on March 13, 2012 and the survey stayed open for three weeks. A second email was sent out after the first week to encourage participation. It was stressed to the recipients that even if they did not pursue an academic or professional career in medical physics, their response was very important.

## III. RESULTS

### A. Participation statistics

The number of applicants, fellows, and average grade point average (GPA) are summarized in Table 2. From 2001 to 2012, there have been 707 applicants and 148 fellowships awarded between the two programs (116 SUFP and 32 MUSE). The analysis of the outcomes and survey feedback include only the 140 fellows who have completed the programs through summer 2011. The 140 fellows consisted of seventy‐five men and sixty‐five women. The number of SUFP and MUSE fellowships awarded is dictated by the AAPM budget. The stipend for both programs has been $4,000 for all years except 2009 and 2010, when it was increased to $4,500. In order to accommodate more fellows, the stipend was reduced back to $4,000 in 2011. The total amount of money dispersed to fellows since the inception of the two programs until summer 2011 is $608,000. The SUFP was able to fund one additional fellow in 2012 due to a donation from the Southern California Chapter of the AAPM. The additional funding is addressed in the Discussion section.

**Table 2:** SUFP & MUSE statistics (inception through 2012).

*Year*	*SUFP Applicants*	*MUSE Applicants*	*SUFP Fellows*	*MUSE Fellows*	*SUFP GPA*	*MUSE GPA*
2001	18	−	6	−	3.77	−
2002	32	−	9	−	3.82	−
2003	44	−	8	−	3.71	−
2004	57	−	10	−	3.85	−
2005	65	−	12	−	3.76	−
2006	63	3	12	3	3.86	−
2007	62	7	13	7	3.82	−
2008	69	10	14	7	3.79	3.32
2009	70	18	14	7	3.88	3.59
2010	60	11	7	4	3.9	3.45
2011	49	5	5	2	3.93	3.44
2012	58	6	6	2	3.88	3.71
Total	647	60	116	32	−	−

The geographic distribution of participating institutions was grouped into regions used by the US Census. There were sixty‐four unique participating institutions. Several institutions hosted fellows multiple times. Institutions from the Northeast, South, Midwest, and West hosted SUFP and/or MUSE fellows 34, 49, 30, and 27 times, respectively. Each time was considered a separate event. The institutions that hosted the most fellows and the number of fellows that they hosted are: University of Pennsylvania (10), Hampton University (8), University of Chicago (6), and Massachusetts General Hospital (6). A complete listing of the host (mentor) institutions is included in Table 3. If an institution hosted more than one fellow, that number is indicated in parentheses.

**Table 3:** Host institutions.

*Region*	*Mentor Institution*	*State*
Midwest (30 Fellows, 16 Institutions)	University of Chicago (6)^(a)^	IL
	Illinois Institute of Technology	IL
	Loyola University Medical Center (2)	IL
	Indiana University (2)	IN
	University of Kansas Medical Center	KS
	Wesley Medical Center	KS
	Henry Ford Hospital	MI
	William Beaumont Hospital	MI
	Mayo Clinic (4)	MN
	University of Minnesota	MN
	Cox Health Systems	MO
	University of Missouri	MO
	Washington University School of Medicine	MO
	Cleveland Clinic (2)	OH
	Marshfield Clinic	WI
	University of Wisconsin (4)	WI
Northeast (34 Fellows, 10 Institutions)	Massachusetts General Hospital (6)	MA
	Memorial Sloan Kettering Cancer Center (5)	NY
	North Shore University Hospital‐ Long Island	NY
	St. Luke's Cancer Center (5)	NY
	SUNY Upstate Medical University	NY
	Geisinger Clinic (3)	PA
	Pennsylvania State University	PA
	Spartanburg Radiation Oncology	PA
	Thomas Jefferson University	PA
	University of Pennsylvania (10)	PA
South (49 Fellows, 22 Institutions)	Lee Moffitt Cancer Center	FL
	MD Anderson Cancer Center Orlando	FL
	University of Florida (2)	FL
	Emory University (3)	GA
	Georgia Institute of Technology	GA
	John D. Cronin Cancer Center (2)	KY
	University of Kentucky (5)	KY
	Louisiana State University	LA
	Johns Hopkins University (3)	MD
	Univ. of Maryland School of Medicine	MD
	Duke University Medical Center	NC
	Richland Memorial Hospital	SC
	St. Jude Children's Hospital (2)	TN
	Vanderbilt University Medical Center (5)	TN
	MD Anderson Cancer Center (4)	TX
	Baylor University Medical Center	TX
	Medical &Radiation Physics, Inc.	TX
	University of Texas HSC San Antonio	TX
	UT Texas HSC	TX
	Hampton University (8)	VA
	University of Virginia (2)	VA
	VA Commonwealth University (2)	VA
West (27 Fellows, 16 Institutions)	University of Arizona	AZ
	Children's' Hospital of LA	CA
	Long Beach Memorial Medical Center	CA
	Therapy Physics, Inc.	CA
	UC San Francisco (5)	CA
	Stanford University Hospital (3)	CA
	UCLA School of Medicine (2)	CA
	Stanford University (3)	CA
	Vail Valley Medical Center	CO
	CAMP	CO
	University of New Mexico (2)	NM
	Good Samaritan Regional Medical Center	OR
	Oregon Health and Sciences University	OR
	University of Utah	UT
	Sacred Heart Medical Center	WA
	University of Washington (2)	WA

a Number in parenthesis indicates how many total fellows participated at that institution.

Projects are encouraged from any subfield of medical physics and may be purely research, purely clinical or combined in nature. On the application, the mentor may designate the proposed topic as therapy, diagnostic or nuclear medicine. Many projects were not identified as belonging to any specific category by the mentor, and projects can change over the course of the summer. Retrospectively we looked at 95 completed projects (for which we had descriptive titles) and grouped them as predominately imaging, imaged guided radiotherapy (IGRT) or predominately therapy (non‐IGRT). Fifty‐four topics were classified as radiation therapy, 28 topics were classified as imaging, and the remaining 13 topics as IGRT.

### B. End of summer evaluations and online survey

Of the 140 SUFP and MUSE participants, 42 fellows and 32 mentors completed End of Summer evaluations. The evaluations for SUFP 2006 and MUSE 2006–2008 years could not be located. Fellows and mentors rated their experience by stating their agreement with various statements on a scale of 1 (completely agree) to 4 (completely disagree). The SUFP and MUSE data are reported together. The overall satisfaction with the programs was very high. The fellows reported the greatest dissatisfaction was the length of the program. Starting in 2003, the fellows were asked if a paper or presentation resulted from their summer experience and the majority (80%) reported yes. The responses to the end of summer surveys are summarized in Table 1.

One hundred and seventeen emails were sent to past fellows inviting them to participate in the online survey. Sixty surveys were completed, yielding an overall return of 51%. Eighty‐three percent of the respondents had participated in the SUFP program and 17% had participated in the MUSE program. The year of participation in the program, total number of fellows that year, and number of survey responses are as follows: 2001 (6 fellows, 1 response), 2002 (9 fellows, 5 responses), 2003 (8 fellows, 1 response), 2004 (10 fellows, 5 responses), 2005 (12 fellows, 5 responses), 2006 (12 fellows, 3 responses), 2007 (13 fellows, 7 responses), 2008 (14 fellows, 12 responses), 2009 (14 fellows, 11 responses), 2010 (7 fellows, 5 responses) and 2011 (5 fellow, 5 responses).

Table 4 summarizes the responses from the 60 past fellows who responded to the online survey. As a result of their participation in MUSE or SUFP, 65% of responders decided to pursue a graduate degree in medical physics. Thirteen fellows pursued an area other than medical physics, and nine fellows reported that their participation in the summer program did not affect their future education choice. At the time of the survey, 16 of the respondents were working in the medical physics profession. The majority of respondents were still in an education program: 7 undergraduates and 26 graduate students or residents. The remaining respondents were not studying or working in the medical physics profession. After the question regarding current education and/or employment status, the survey was completed for respondents who reported that they were no longer involved with medical physics. The remaining three questions regarding board examination, AAPM, and publications were only asked of those currently engaged in medical physics.

**Table 4:** Summary of online survey of past fellows.^a^

*Question*	*Response*
Fellow decided to pursue a medical physics graduate degree as a result of participation in SUFP or MUSE	39 (65%)
Currently involved in medical physics as a graduate student, resident or professional	42 (70%)
Of those who are currently involved in medical physics (42 past fellow):
ABR, CCPM or ABMP board certified or in process of certification	31 (74%)
Member of AAPM	33 (79%)
Published, or are in the process of, publishing research or clinical work	29 (69%)

a 117 survey requests emailed, 60 respondents.

Regarding board examinations administered by the American Board of Radiology (ABR), Canadian College of Physicists in Medicine (CCPM) or American Board of Medical Physics (ABMP), only three of respondents were board‐certified, but 28 were in the certification process. The remaining respondents were not intending to take the exam because it was not applicable to their current discipline. Respondents were asked about their current level of involvement in the AAPM. The majority are either student, junior, full or corporate members. Only three past fellows are involved on committees. Ten fellows reported that they are engaged in medical physics but not involved with the AAPM. Respondents were asked about their publications and presentations in the field of medical physics. The vast majority has presented their work either in a published journal or oral/poster presentation.

## IV. DISCUSSION

### A. Demographic data and statistics

The AAPM has been supporting undergraduate summer fellowships with the SUFP and MUSE programs since 2001 and 2006, respectively. From 2001 to 2012, 148 fellowships have been awarded and a total of $608,000 has been dispersed to fellows. The first objective of the paper was to compile and analyze the demographic data since 2001 to report on the programs' history. It is clear that interest in the programs has grown, as seen by the increase in number of applicants until its peak (88 applicants) in 2009. This trend is in line with the overall growth of the field of medical physics, as seen by increased applicants in graduate programs, number of programs, and number of applicants sitting for the board exams. The decline in applicants from its peak is assumed to be a natural leveling out following a period of rapid growth in the medical physics field. Following the peak, the total number of applicants in 2010, 2011, and 2012 were 71, 54, and 64, respectively. Despite this small decline, the caliber of applicants remained very high. Throughout the history of the programs, the average GPA of SUFP and MUSE fellows are 3.83 and 3.50, respectively, and have remained very high, as illustrated in Table 2. The total number of fellowships granted since its peak in 2008 and 2009 was reduced in 2010 due to funding cuts to SUFP and MUSE as a result of overall reductions in the AAPM budget during the global financial crisis of that era.

The gender of the fellows yielded an interesting result. The percentage of women (46%) is notable considering how few women are in physics undergraduate programs. According to American Institute of Physics, less than 21% of undergraduate physics degrees were awarded to women in 2010. We believe the higher number of women is in line with other medical areas such as pharmacy, veterinary science, and medical science, where women are the predominate gender. However, the higher percentage of women fellows in SUFP and MUSE is not reflected in the ratio of students entering graduate medical physics programs. According to SDAMPP, the ratio of men to women in medical physics graduate programs was 71 to 29 in 2009 and 70 to 30 in 2010, respectively. The geographic distribution of hosting institutions illustrated significant participation from all areas of the US and at many distinct institutions. Many fellows identify specific project types and/or geographic location preferences, so it is beneficial to maintain a large pool of potential mentors spread across the continent.

### B. Participant satisfaction

In addition to demographic data over the course of the programs, the authors looked at participant satisfaction. Results from the End of Summer surveys indicate that the vast majority of both fellows and mentors were very pleased with the program. This high level of satisfaction agrees with studies of undergraduate research experience in other fields. Fellows found their research and/or clinical experience very rewarding and would consider a career in medical physics. The fellows and mentors were pleased with their interaction with AAPM during the application process. Assistance with housing received a lower ranking, a 1.7 out of 4. The summer housing situation at each institution is obviously different and it has proven difficult for the AAPM to help mentors assist fellows in this regard. The mentors are encouraged to work with their institutions to help with housing to the best of their ability. Fellows expressed some dissatisfaction (average ranking of 3.2 out of 4) with the length of the program (responding that they either found the program too long or too short). The program is ten weeks, which agrees with the majority of other summer undergraduate programs and the practical length of time between regular semesters for many undergraduates.

The responses from the mentor survey indicated a similar positive experience, with the most difficult task being assisting the fellow with housing. High satisfaction was reported in integrating students into research, the work output, and the desire to mentor a fellow in the future. Positive mentor and fellow experiences are important for three reasons. First, having a good experience motivates and encourages the fellow to continue in this field. Secondly, the mentor has the satisfaction of making progress on their project, and in fact, a publication, presentation or an abstract was the outcome for a majority of participants. Lastly, when mentors share feedback about positive experience amongst their peers, it may encourage other potential mentors to participate in future years. The authors hope that reporting on positive feedback from both fellows and mentors will stimulate other medical physicists to join the effort. This can only further strengthen the programs.

### C. Effect on graduate education choices

The efficacy of the programs was assessed using four metrics: entry into a medical physics graduate program, board certification, publications, and AAPM involvement. This study is limited to tracking if, and how, the summer programs affect the fellows graduate education choices, not their ultimate career path. The online survey of past recipients provides a quantitative look at the educational outcome of the programs. There were 60 respondents. The most compelling result is that 65% of those responding decided to pursue medical physics for graduate education as a result of their participation in the program. However, this represents roughly 28% of total past fellows. We had no way of tracking what non‐responding fellows chose to do. Anecdotally, the authors have heard that several fellows went on to medical school. While this did not achieve the stated objectives of the program to encourage entry into medical physics, it is a “second order success” in that it hopefully creates physicians who appreciate the discipline of medical physics.

The respondents were asked about their present education or employment status. Note that in the survey, current education, and/or employment status was used as a surrogate for entry into a medical physics graduate program. Seven respondents were still undergraduates. Twenty‐six respondents were enrolled in a medical physics graduate or residency program. Sixteen respondents are employed as medical physics professionals. Only 11 of the respondents were not studying or working in medical physics. Past SUFP and MUSE recipients are asked about their ABR certification process participation in the survey as an additional metric of medical physics involvement. However, it is important to note that there are many medical physics graduates working in fields such as industry and academia that do not require board certification. We also recognize that since the programs are still young, even the early participants may still be in graduate school pursuing doctorates or in residency programs. Therefore, not many (only three respondents) are board‐certified. However most (67% of respondents engaged in medical physics) are in the process of board certification. This means that of the respondents that did choose medical physics profession, most are pursuing board certification or are board‐certified.

Most respondents (79%) are involved in AAPM. This is a success in that we not only want to encourage graduate school in medical physics but also involvement in AAPM. Further supporting the success of the program is the high level of self‐reported participation in publications and presentations. Based on the survey, 69% have published or are in the process of publishing work in medical physics. This indicates that fellows are continuing on a trajectory of success.

### D. Suggestions for future enhancements

We also conclude that the programs' success and participation are limited primarily by the availability of AAPM funds. The number and quality of fellowship applicants is very high and if there were more funds, additional excellent undergraduates would benefit along with the long‐term health of the discipline. It is suggested that the MUSE and SUFP committees aim to solicit funds from additional sources. A prime example of outside support (not from the national AAPM) is a $4,000 donation from the Southern California AAPM Chapter. The funds were used to support one additional fellow for the 2012 summer. This donation will be highlighted in the AAPM newsletter and it is hoped other chapters contribute in a similar fashion. However, chapter funds are limited as well, and perhaps a targeted campaign to past fellows for donations would be a useful future fundraising strategy.

The subcommittees have considered merging the two programs. A reduction of administrative overhead would be an advantage for both the MUSE and SUFP programs. A potential disadvantage for MUSE could be a loss of focus and attention to minority student‐specific needs and recruitment. MUSE may benefit from a wider geographic distribution of participating institutions. A decision has not been reached yet regarding merging the programs. Lastly, given the difficulty tracking past fellows, we advocate creating a more robust database for past fellows and project topics. Throughout the tenure of the programs, the only method used to collect current contact information of the fellows was from the End of Summer evaluations. Less than half the participants completed the surveys. A database would help not only track the success of the program, but also maintain contact with past fellows to recruit their participation on education committees and as a possible source for future charitable contributions. Development of an online application submission system is also encouraged.

## V. CONCLUSIONS

The authors conclude that the AAPM sponsored MUSE and SUFP programs are an effective way to introduce and steer high‐achieving physics undergraduates into the field of medical physics. Recruiting top undergraduates into medical physics to meet the projected demand should remain a priority. Practical experience in medical physics during their undergraduate tenure is an important tool in attracting students into this graduate field. The programs have provided a useful and rewarding experience for both mentors and fellows, and are the only undergraduate research programs specific to medical physics. Despite the need to decrease the number of awards due to economic realities, the program is popular and should continue. An improved database for tracking fellowship recipients is warranted. Additional funding streams should be pursued. The possibility of combining the MUSE and SUFP programs is currently being discussed.

## ACKNOWLEDGMENTS

The authors would like to thank Ms. Jackie Ogburn, AAPM Education Manager, for her tireless work with both the SUFP and MUSE programs and this current project. We appreciate all her assistance tracking down the data and managing the online survey. The authors would also like to acknowledge the outstanding efforts of all the past and current volunteer members of the SUFP and MUSE AAPM subcommittees.
